# Blue Cone Monochromacy: Visual Function and Efficacy Outcome Measures for Clinical Trials

**DOI:** 10.1371/journal.pone.0125700

**Published:** 2015-04-24

**Authors:** Xunda Luo, Artur V. Cideciyan, Alessandro Iannaccone, Alejandro J. Roman, Lauren C. Ditta, Barbara J. Jennings, Svetlana A. Yatsenko, Rebecca Sheplock, Alexander Sumaroka, Malgorzata Swider, Sharon B. Schwartz, Bernd Wissinger, Susanne Kohl, Samuel G. Jacobson

**Affiliations:** 1 Scheie Eye Institute, Department of Ophthalmology, Perelman School of Medicine, University of Pennsylvania, Philadelphia, Pennsylvania, United States of America; 2 Hamilton Eye Institute, Department of Ophthalmology, University of Tennessee Health Science Center, Memphis, Tennessee, United States of America; 3 Pittsburgh Cytogenetics Laboratory, Center for Medical Genetics and Genomics, University of Pittsburgh School of Medicine, Pittsburgh, Pennsylvania, United States of America; 4 Molecular Genetics Laboratory, Institute for Ophthalmic Research, Centre for Ophthalmology, University of Tuebingen, Tuebingen, Germany; Hadassah-Hebrew University Medical Center, ISRAEL

## Abstract

**Background:**

Blue Cone Monochromacy (BCM) is an X-linked retinopathy caused by mutations in the *OPN1LW / OPN1MW* gene cluster, encoding long (L)- and middle (M)-wavelength sensitive cone opsins. Recent evidence shows sufficient structural integrity of cone photoreceptors in BCM to warrant consideration of a gene therapy approach to the disease. In the present study, the vision in BCM is examined, specifically seeking clinically-feasible outcomes for a future clinical trial.

**Methods:**

BCM patients (n = 25, ages 5–72) were studied with kinetic and static chromatic perimetry, full-field sensitivity testing, and eye movement recordings. Vision at the fovea and parafovea was probed with chromatic microperimetry.

**Results:**

Kinetic fields with a Goldmann size V target were generally full. Short-wavelength (S-) sensitive cone function was normal or near normal in most patients. Light-adapted perimetry results on conventional background lights were abnormally reduced; 600-nm stimuli were seen by rods whereas white stimuli were seen by both rods and S-cones. Under dark-adapted conditions, 500-nm stimuli were seen by rods in both BCM and normals. Spectral sensitivity functions in the superior retina showed retained rod and S-cone functions in BCM under dark-adapted and light-adapted conditions. In the fovea, normal subjects showed L/M-cone mediation using a 650-nm stimulus under dark-adapted conditions, whereas BCM patients had reduced sensitivity driven by rod vision. Full-field red stimuli on bright blue backgrounds were seen by L/M-cones in normal subjects whereas BCM patients had abnormally reduced and rod-mediated sensitivities. Fixation location could vary from fovea to parafovea. Chromatic microperimetry demonstrated a large loss of sensitivity to red stimuli presented on a cyan adapting background at the anatomical fovea and surrounding parafovea.

**Conclusions:**

BCM rods continue to signal vision under conditions normally associated with daylight vision. Localized and retina-wide outcome measures were examined to evaluate possible improvement of L/M-cone-based vision in a clinical trial.

## Introduction

Blue cone monochromacy (BCM) is a congenital retinal disorder caused by mutations in the *OPN1LW* / *OPN1MW* gene cluster on the X chromosome that controls the expression of the red (L, long wavelength) and green (M, middle wavelength) cone photoreceptor opsins. The genes that express the opsin for the third cone subtype, S- (short wavelength) or blue cones, and the rod pigment are autosomal and not involved in BCM [1–4].

There is a long history of investigation of the visual abnormalities in BCM. It is known that L/M cone vision in BCM is either severely abnormal or not detectable, and rods and S cones dominate perception [2,4–14]. Little has been known, however, about the micro-structural integrity of the retina in BCM and specifically the existence of L/M cone photoreceptors [4,15,16]. Recently, it was questioned whether there was evidence of L/M cone photoreceptors in the retinas of a cohort of BCM patients with cone opsin gene array mutations that would predict a lack of red or green opsin expression. *In vivo* cross-sectional and *enface* retinal imaging in this BCM cohort revealed that there were abnormalities of retinal lamination and photoreceptor mosaic disruption. The foveal region showed thinning of the photoreceptor outer nuclear layer and shortening of cone outer segments. Yet, there were residual L/M-cones within the central 1.5 mm of the retina. This retinal structural evidence indicated that BCM warrants consideration for human clinical trials of L/M-cone gene therapy [4].

The next step is taken in the current study which establishes the range of visual function results in a cohort of BCM patients and takes the first steps to decide which clinically-feasible outcome measures, in addition to the ocular examination, would be useful to provide evidence of safety and efficacy in a clinical trial that aims to improve L/M-cone function.

## Materials and Methods

### Human Subjects and Ethics Statement

Human studies were approved by the Institutional Review Boards at the University of Pennsylvania (700942 and 191700) and the University of Tennessee Health Science Center (98-06657-FB). For adult subjects, written informed consent was obtained. For all children, written parental permission was obtained. Written assent was obtained from children ages 12 to 17; oral assent was obtained from children ages 7 to 11; children under the age of 7 years were enrolled with written parental permission. The procedures adhered to the tenets of the Declaration of Helsinki. There were 25 patients, representing 15 families, with BCM, diagnosed clinically and by molecular genetics (S1 Table). All subjects underwent a complete eye examination.

### Perimetry

Kinetic visual fields were tested with a Goldmann perimeter (V-4e stimulus). Two-color dark-adapted static threshold perimetry was performed with 500-nm and 650-nm stimuli, and light-adapted perimetry was studied with 600-nm and achromatic (white) stimuli on standard 10 cd.m^-2^ white backgrounds [17,18], and with a 440-nm stimulus on a 100 cd.m^-2^ yellow background [19–21]. Stimuli were Goldmann size V (1.7° diameter) and 200 ms in duration; the 440-, 500-, 600- and 650-nm stimuli had a bandwidth of 10 nm. The stimuli were presented along the vertical meridian extending 30° superior and inferior from fixation. Photoreceptor mediation under the dark-adapted condition was determined from the difference between 500-nm and 650-nm sensitivities [17,18]. The estimates of photoreceptor mediation under light-adapted conditions, on the other hand, were more complex and were inferred from differences in relative effectiveness of 440-nm, 600-nm and achromatic targets in stimulating rod and cone photoreceptors.

### Spectral Sensitivity Function

Spectral sensitivity functions were measured in normal subjects (n = 3, ages 23–30) and in a subset of 8 BCM patients using a modified perimeter with methods previously described [4,22–25]. In brief, the test stimulus (1.7° diameter, 200-ms duration) was spectrally shaped by interference filters and presented at 14° superior to fixation. Thresholds are reported normalized to energy. Spectral sensitivity functions for rods, S- and L/M- cones are also shown in term of relative energy. Spectral sensitivity functions were recorded under dark-adapted conditions and on a standard 10 cd.m^-2^ white background. Sensitivities were fit with scotopic and photopic luminosity functions, and an S-cone spectral sensitivity function [26–28].

### Full-field Stimulus Testing (FST)

FST was used to obtain psychophysical thresholds with blue (peak 465 nm) and red (peak 637 nm) stimuli in the dark-adapted state and on white and chromatic backgrounds. Techniques for performing and analyzing results of FST are published [18,25,29].

### Eye Movement and Fixation Monitoring with Retinal Imaging

Eye movements were recorded by video imaging (25 Hz) the retina under near-infrared (NIR) illumination invisible to the subject with a microperimeter (MP1, Nidek Technologies America, Inc, Greensboro, NC, USA) using methods previously described [30–32]. Patients fixated to a continuously-illuminated target in a dark room with a black curtain blocking any stray light from reaching the subject’s eye. The fixation target subtended ~1° in the visual field. The optical coherence tomography (OCT) derived location of the foveal depression was used to define the exact location of the anatomical fovea. An overall fixation instability measure based on the upper limit (mean+2SD) radial extent was calculated over each 10 s long epoch.

### Chromatic Microperimetry with Retinal Tracking

Visual function at the anatomical fovea and parafoveal region was evaluated using microperimetry with retinal tracking. In order to better understand contributions of L/M cones and S cones, two test conditions were used: blue stimuli on a yellow background (BonY) provided relatively greater opportunity to detect S-cone function and red stimuli on cyan background (RonC) provided relatively greater opportunity to detect L/M-cone function. Chromatic stimuli and backgrounds were achieved by editing the calibration table used by the instrument. Stimuli were Goldman V sized and 200 ms long. A custom test pattern densely covering the foveal region and extending to parafoveal region along the four major meridians was used. In majority of BCM patients, the anatomical location of the fovea was defined based on OCT scans and transferred to the microperimeter with the ‘OCT on Fovea’ function. Subjects fixated at the center of a six-degree diameter ring in order to evaluate the foveal region. Thresholds were exported and analyzed.

## Results

### Clinical and Molecular Characteristics of the BCM Cohort

A cohort of 25 patients with BCM was studied (S1 Table; patient numbers correspond to those used in our previously published structural data [4] to allow comparisons). The genetic analyses identifying deletions of various extents at the *OPN1MW* / *OPN1LW* gene cluster in 16 of the patients, representing 9 families, have been previously reported (P1-P10; P15-20) [4]. Among the remaining 9 patients, there were 4 patients (2 families) also with deletions at the *OPN1MW / OPN1LW* gene cluster; 2 patients (2 families) with deletion of the LCR only; 1 patient with a single red-green hybrid gene with the C203R point mutation; and 2 patients with the deleterious C203R point mutation in at least one gene of the *OPN1MW / OPN1LW* gene cluster [33,34]. Best-corrected visual acuities ranged from 20/63 to 20/200 and there was a range of refractive errors from -0.50 to -12.75D (mean, -6.50D; SD, 3.32D). Spectral-domain OCT results from 8 of the 9 previously unreported patients were available and these were analyzed as in our previous report [4]. The results indicated that in 7 of these 8 patients, outer nuclear layer (ONL) thickness was within normal limits across most of the scan except in a central region of abnormal thinning. The oldest patient, age 72, had ONL thinning beyond the central region (S1 Fig).

### Visual Function under Light- and Dark-Adapted Conditions

Goldmann kinetic perimetry, a traditional outcome measure performed under light-adapted conditions to assess vision mediated by L/M cones, uses a photopic 10 cd.m^-2^ white background light that desensitizes rod-based vision [35,36]. In BCM, given the L/M-cone deficiencies, it would be hypothesized that there could be abnormal test results. In the cohort of BCM patients with kinetic fields available (n = 22), there were 10 patients with a normal extent (V-4e, achromatic target); in 12 of the patients, the fields were generally full but mildly abnormal (percent of normal extent, mean = 81%, range 62–88%) [37]. These results support the hypothesis that a normal extent of kinetic field with this target size and intensity does not indicate normal functioning L/M-cones in BCM and confirm previous reports [9,10,38].

**Fig 1:**
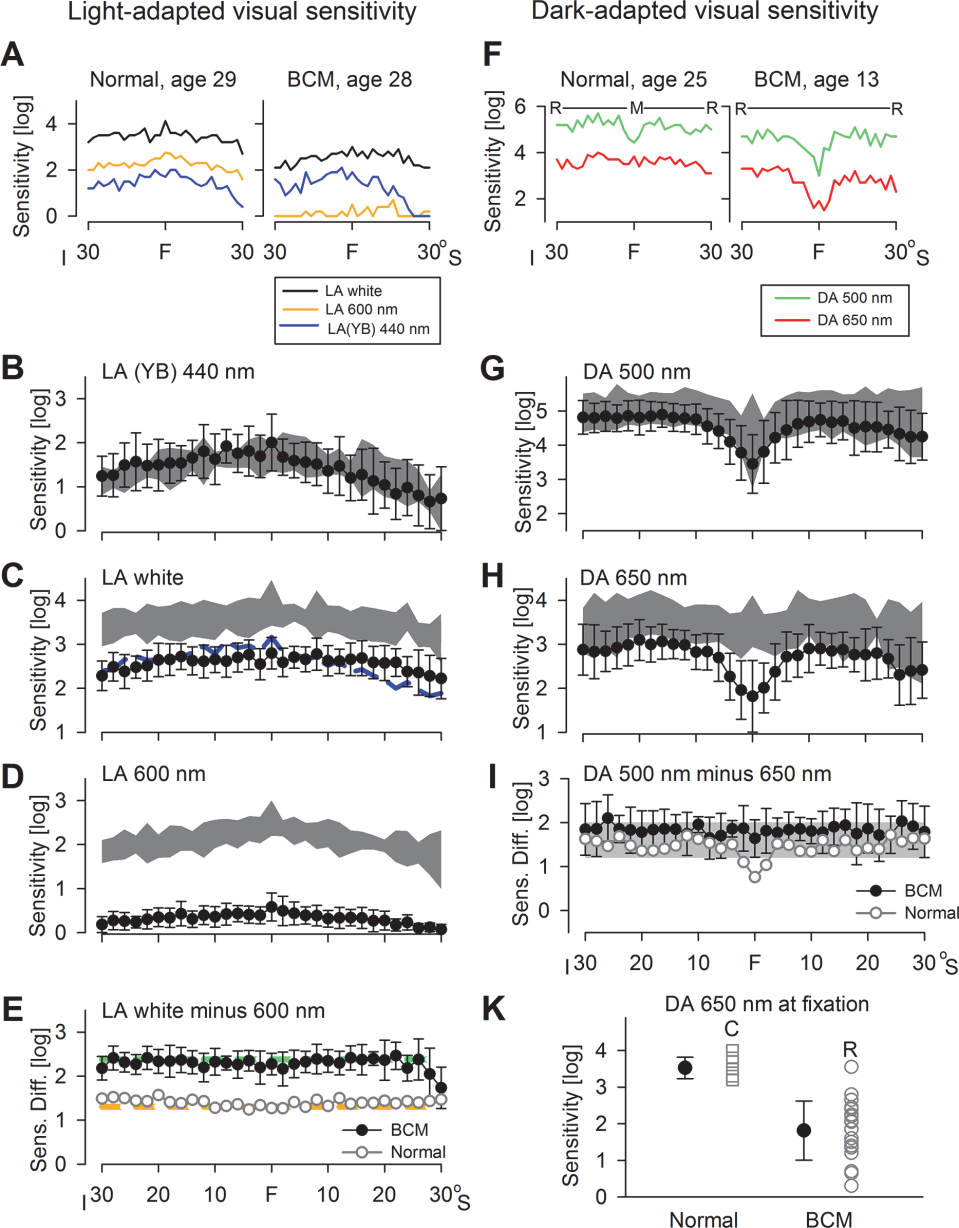
Visual fields of BCM patients evaluated with kinetic and static perimetry. (A) Light-adapted (LA) vertical sensitivity profiles from a normal subject and a BCM patient using achromatic (black line) and 600-nm (orange line) stimuli on a 10 cd.m^-2^ white background, or 440-nm (blue line) stimuli on a yellow background (YB). (B) S-cone sensitivity profiles (filled circles) of the BCM patients using a 440-nm stimulus on YB compared to normal limits (gray = ±2SD). (C) LA white vertical sensitivity profiles of BCM patients (filled circles) compared to normal (gray). Blue line is the S-cone sensitivities from Panel C shifted according to the difference in effectiveness between the white and 440-nm stimuli. (D) LA 600-nm vertical sensitivity profiles of BCM patients (filled circles) compared to normal (gray). (E) Sensitivity differences between LA white and LA 600-nm stimuli are shown for the BCM patients (filled circles) and normal (unfilled circles). Predicted differences for rod (green dashes) and L/M cone (orange dashes) mediation are shown. (F) Dark-adapted (DA) vertical sensitivity profiles from a normal subject and a BCM patient using 500-nm (green line) and 650-nm (red line) stimuli. Above the results it is shown whether there is rod (R) or mixed (M) mediation, as determined by the differences between sensitivities to the stimuli. (G) DA 500-nm vertical sensitivity profiles of BCM patients (filled circles) compared to normal (gray). (H) DA 650 nm vertical sensitivity profiles of BCM patients (filled circles) compared to normal (gray). (I) Sensitivity differences between DA 500- and DA 650-nm stimuli are consistent with rod mediation (gray) at all locations except for the normal results with 650 nm at fixation. S, superior; I, inferior. (J) DA 650-nm sensitivities at fixation in normal and BCM. Normal 650-nm sensitivities are mediated by the L/M cones (C) whereas BCM sensitivities are mediated by the rods (R). Error bars are ±1SD.

Visual sensitivities were measured with static perimetry in the light-adapted state with three different stimuli (Fig 1A–1E) and in the dark-adapted state with two stimuli (Fig 1F–1J). Testing was performed along the vertical meridian through fixation, spanning 60°. Sensitivities using white and 600-nm stimuli on a 10 cd.m^-2^ white background, and 440-nm stimuli on a 100 cd.m^-2^ yellow background are shown for a representative normal subject and a BCM patient (Fig 1A). This comparison indicates that 440-nm sensitivities are qualitatively similar between normal and BCM, whereas sensitivities to 600-nm and white stimuli are lower in the BCM patient than in the normal subject.

S-cone-specific function was measured with 440-nm stimuli on a yellow background, and the results from the cohort of BCM patients (n = 17) were within normal limits (Fig 1B). Normally, sensitivity to white (Fig 1C) or 600-nm (Fig 1D) stimuli on white standard backgrounds are mediated by L/M-cones. BCM patients (n = 21) tested under these conditions showed significant reductions in sensitivity at all tested locations (Fig 1C and 1D). Notably, the sensitivity loss to white stimuli (average = 0.94 log units) was substantially smaller than the sensitvity loss to 600-nm stimuli (average = 1.78 log units). These results implied that different photoreceptor mechanisms were contributing to vision under these daylight conditions depending on the stimulus.

To evaluate the hypothesis that S-cone function was driving the results with white stimuli on the white background, S-cone function estimated with 440-nm stimuli on a yellow background were extrapolated to white stimuli on the white background (Fig 1C, blue line) assuming S-cone sensitivities to be equal for both adapting conditions. Extrapolated S-cone function could explain the profiles with white stimuli in the inferior visual field. In the superior visual field, however, there was a small but consistent difference in average sensitivity supporting the contribution of a non-S-cone mechanism. Sensitivities to 600-nm stimuli were ~3–4 log units higher than what would be predicted from S-cone function. Non-S-cone mechanisms in BCM would potentially include either rod function desensitized by the background or remnant L/M-cone function. The average sensitivity difference between white and 600-nm stimuli in normal subjects (1.41 log) was similar to that expected (1.31 log) if both were mediated by L/M cones (Fig 1E). Whereas the sensitivity difference in BCM (2.38 log) was equal to that expected (2.38 log) if both were mediated by (light-adapted) rods (Fig 1E).

Dark-adapted sensitivities of a normal subject and a representative BCM patient along the vertical meridian are illustrated (Fig 1F). The 500-nm and 650-nm sensitivity profiles from normal subjects are known to be rod-mediated except in the central few degrees [17,18]. Centrally, there is mixed mediation with detection of 500 nm by rods and of 650 nm by L/M-cones even after accounting for the contribution (~0.3 log at 500 nm) of macular pigment. The main difference in the BCM profile compared to normal was that there was rod mediation across the entire profile and a central depression with the 650-nm profile.

Dark-adapted data from BCM patients were compared with those of normal subjects for the 500-nm and 650-nm stimuli. The average 500-nm sensitivity profile in BCM (n = 24) was either at the lower end of the normal range or slightly below it (Fig 1G). There was no apparent relationship of mean 500-nm sensitivity to age (r^2^ = 0.003). The average 650-nm profile in BCM (n = 20) differed from the normal 650-nm profile in the central ~10° diameter region with the greatest difference being at or near fixation; beyond 6° eccentricity, most BCM sensitivities were normal or near normal (Fig 1H). The difference between 500-nm and 650-nm sensitivities in BCM varied little from the predicted sensitivity difference for rod-mediated vision along the vertical meridian, which is 1.6 (±0.4) log for the stimuli used (Fig 1I).

Next, BCM vision at fixation was evaluated in greater detail. The dark-adapted sensitivity to a 650-nm stimulus at fixation (1.81±0.81 log; n = 19) was significantly lower than normal (3.53±0.29 log; n = 8); 1.71 log difference provided a large dynamic range over which potential improvements could be detected (Fig 1J). The difference between 500-nm and 650-nm sensitivities at fixation showed cone mediation in normal subjects but rod mediation in BCM patients.

### Photoreceptor contributions across a wide spectrum of wavelengths

**Fig 2:**
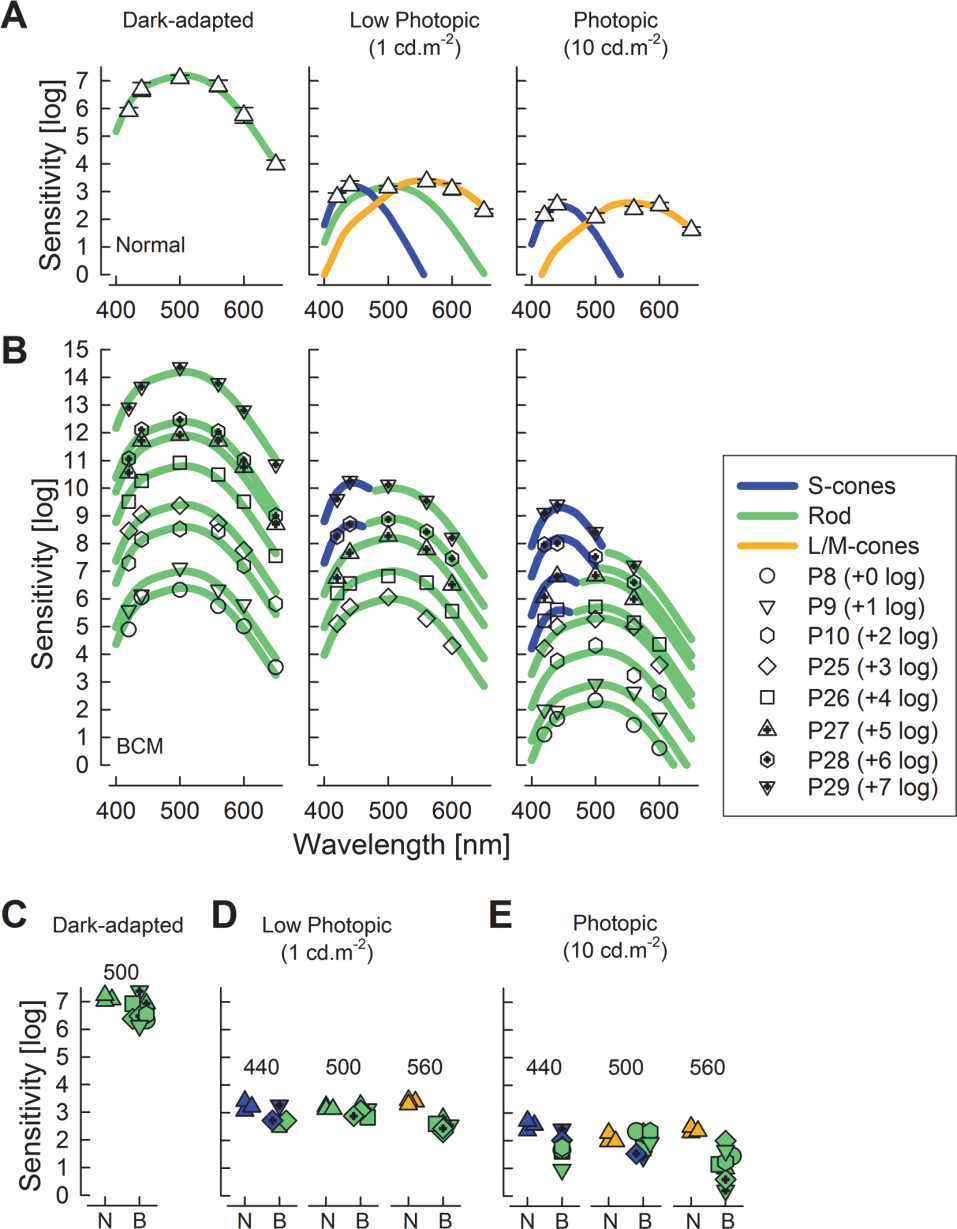
Spectral sensitivity functions in normal subjects and BCM patients recorded at 14° superior field. (A) Sensitivities (mean±1 SD) to six spectrally distinct stimuli in normal subjects (n = 3) under dark-adapted (left), and on 1 (middle) and 10 cd.m^-2^ (right) white backgrounds. (B) Sensitivities to the spectrally distinct stimuli in BCM patients for the same three adaptation conditions as in Panel A. Results from P8 are shown at the correct ordinate location; results from remaining patients have been adjusted by 1 log increments for visibility. Theoretical functions describing rod (green), S cone (blue), L/M cone (orange) sensitivities are shown after vertical shifts to fit relevant normal and BCM data in Panels A and B. (C) Comparison of individual normal and BCM sensitivities at 500 nm. (D,E) Comparison of individual normal and BCM sensitivities at 440, 500 and 560 nm. Symbols in Panels C, D, and E are painted by colors derived from the fit of theoretical functions to the spectral data. N = normal, B = BCM.

To better understand the photoreceptor origin of visual function in BCM in the superior field where perimetric data implied existence of non-S-cone mechanisms under light-adapted conditions, the sensitivity at 14° superior field was evaluated using six wavelengths (Fig 2). In normal eyes at this superior field location, under dark-adapted conditions, all stimuli are seen by rod photoreceptors (Fig 2A, left panel). A 1 cd.m^-2^ white background, reduces the sensitivity by 3 to 4 log units (depending on wavelength) and mediation becomes complex: shorter wavelengths are seen by S-cones, longer wavelengths by L/M-cones, whereas near 500 nm there is evidence for rod vision (Fig 2A, middle panel). With an increase in the white background light to 10 cd.m^-2^, normal vision is describable by the combination of S- and L/M-cone vision (Fig 2A, right panel) as would be expected from this standard photopic adaptation level.

Sensitivity at 14° superior field in BCM patients (P8-P10,P25-P29) under dark-adapted conditions is mediated by rods across all tested wavelengths (Fig 2B, left panel). BCM sensitivities at 500 nm tended to be 0.5 log lower on average (Fig 2C) but the difference was not statistically significant (t-test, P = 0.09). Under low photopic (1 cd.m^-2^ white) conditions (available for P25-P29), at the tested superior field location, some BCM patients showed evidence of S-cone function at shorter wavelengths, whereas S-cone function was not detectable above rod function in others (Fig 2B, middle panel) consistent with a relative S-cone asymmetry apparent along the vertical meridian (Fig 1B). When S-cones were detectable at 440 nm, their sensitivity was not different than normal (Fig 2D). At 500 nm, sensitivities in normal subjects and in BCM patients were mediated by rods. At longer wavelengths however, sensitivities in normals were mediated by L/M cones whereas in BCM the sensitivities were mediated by rods (Fig 2B, middle panel).

Under standard photopic 10 cd.m^-2^ white adapting conditions, half of the tested BCM patients showed S-cone function at shorter wavelengths; others had only rod function detectable (Fig 2B, right panel). When detectable, S-cone function in BCM was near normal sensitivity (Fig 2E). Beyond 500 nm, BCM patients showed spectral evidence of only rod function (Fig 2B, right panel).

It is important to emphasize that 560 nm stimuli under rod-desensitizing adapting conditions showed apparent sensitivity losses of 0.85 log (Fig 2D) or 1.2 log (Fig 2E) compared to normal. These losses may superficially be thought to represent remnant L/M cone function; however, spectral sensitivity functions showed that the apparent losses resulted from differences in photoreceptor mediation, and thus underestimate the true loss of L/M cone function, which was not measurable at this retinal location in these BCM patients.

### Defining Photoreceptor Mediation in BCM at Different Ambient Illuminances

The perimetric and spectral finding that daylight vision (as evaluated at the standard 10 cd.m^-2^ white background) in BCM was at least partially mediated by rods prompted testing of the hypothesis that photoreceptor mediation at higher ambient illuminances could uncover remnant L/M cone function. In order to extend the dynamic range available, simplify the methodology, and avoid issues with fixation instability, FST was used with chromatic stimuli on a wide range of white and chromatic backgrounds; to evaluate the increasing suppression of rod signals, the threshold-versus-intensity (TVI) paradigm was employed [18,23–25,29].

**Fig 3:**
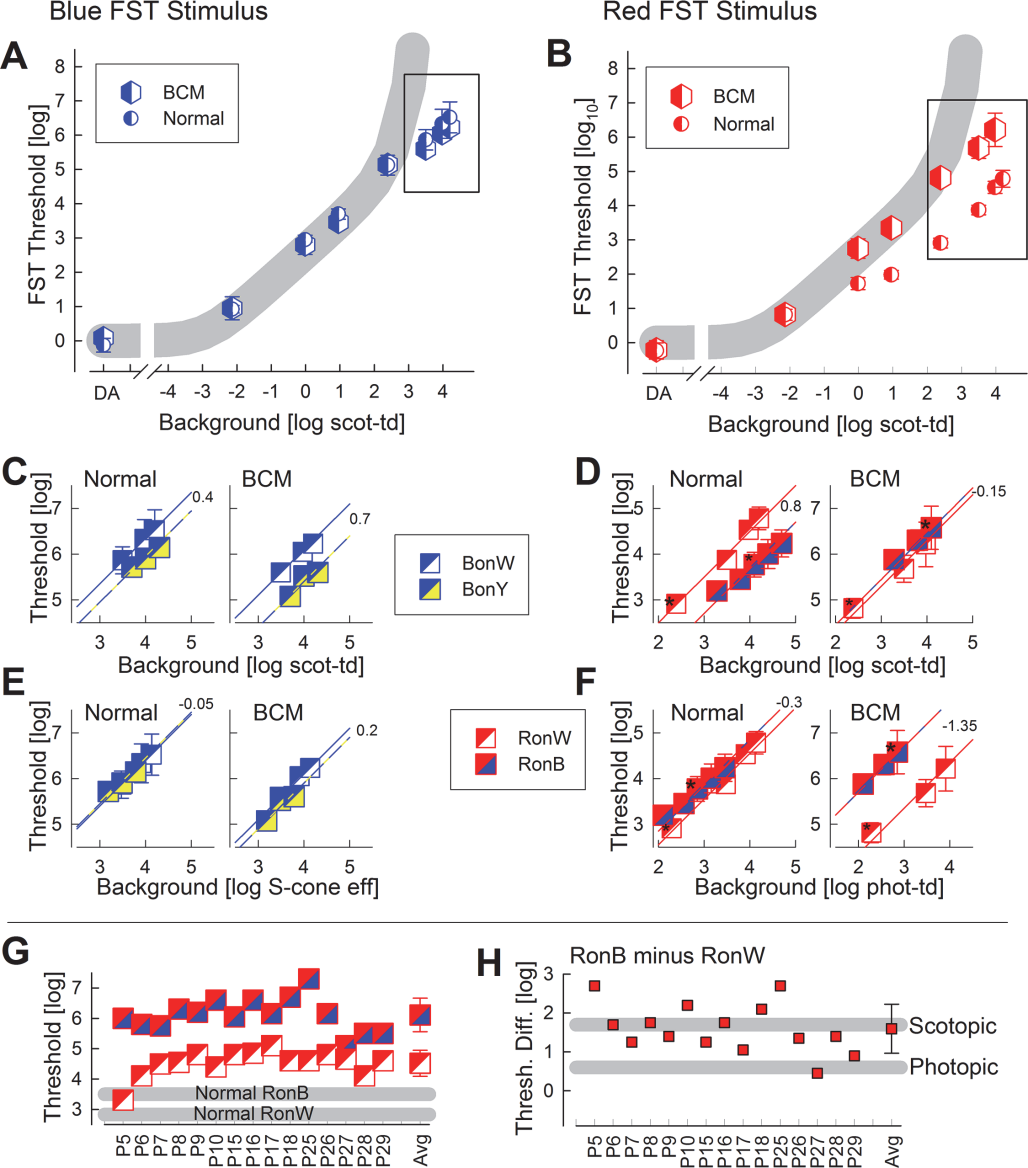
FST results with two colors under dark-adapted conditions and on a range of white and chromatic backgrounds. (A,B) FST-TVI with blue (A) and red (B) stimuli on white backgrounds in BCM patients and normals. Gray region defines the expected desensitization of the rod system. Rectangles define the data further explored in panels C-F. (C-F) Comparison of thresholds with chromatic and white backgrounds; blue stimuli on white (BonW) or yellow (BonY), and red stimuli on white (RonW) or on blue (RonB) backgrounds are shown. Different panels show the effectiveness of the background for the scotopic (C,D), S-cone (E) and photopic (F) systems. Lines with unity slope are fit to the data, and offset between the lines is shown in log units. (G,H) Sensitivity loss and predicted photoreceptor mediation using a pair of RonW and RonB FST thresholds. Both individual results and group averages (Avg) are shown. Error bars, when visible, are ±1SD.

In BCM patients (n = 5) and normal subjects (n = 3), sensitivity to blue full-field stimuli under dark-adapted conditions or on a range of white backgrounds up to ~2 scot-td followed the TVI curves expected from incremental desensitization of rods [25]. Above 2 log scot-td, both BCM and normal thresholds continued along a linear trajectory on log-log coordinates (Fig 3A) whereas underlying rod function would be expected to saturate [25]. When using a red full-field stimulus, BCM and normal thresholds are driven by rods under dark-adapted conditions and on very dim background lights (Fig 3B). In normal subjects, thresholds deviate substantially from the expected rod curve as the L/M-cone mediated vision takes over beyond -1 log scot-td. BCM thresholds with the red stimulus, on the other hand, continued to follow along the curve expected from incremental desensitization of rods. Above 2 log scot-td, BCM thresholds continued along a linear trajectory on log-log coordinates (Fig 3B).

In order to better understand the photoreceptors mediating vision under high ambient illumination conditions, additional experiments were performed with chromatic backgrounds. Thresholds to blue stimuli on white backgrounds (BonW) were compared to those on yellow backgrounds (BonY) by considering the differences in efficiency to stimulate rods (Fig 3C), S-cones (Fig 3E), and L/M-cones (not shown). All sets of data were explained with thresholds rising approximately with unity slope on log-log coordinates as a function of background luminance. In normals, the consistency between white and yellow backgrounds resulting from equating them for S-cone effectiveness (Fig 3E, left), but not for rod (Fig 3C, left) or L/M-cones (not shown), suggested a dominant S-cone component contributing to the visibilty of the full-field blue stimulus under these high ambient illumination conditions. Results for BCM (Fig 3C, right; Fig 3E, right) were comparable to those for normals.

Thresholds to red stimuli on white backgrounds (RonW) were compared to those on blue backgrounds (RonB) by considering the differences in efficiency to stimulate rods (Fig 3D) and L/M-cones (Fig 3F). S-cone function was not considered with red stimuli. For normal subjects, perception of full-field red stimuli was most consistent with photopic L/M-cone mediation (Fig 3F, left), whereas for BCM patients, red stimuli were seen by the scotopic rod system (Fig 3D, right).

### Full-field Efficacy Outcome Measure in BCM

Based on the detailed FST results obtained with two stimuli on a range of white and chromatic backgrounds in a subset of BCM patients, it was hypothesized that a pair of red FST thresholds could be used as potential outcome measures. One of the measures chosen was RonW FST on a standard 2.3 phot-td (2.4 scot-td) white background, and the other was a RonB FST on a 2.9 phot-td (4.1 scot-td) blue background (RonB); these measures were tested in a larger cohort of BCM patients (n = 15). In normal subjects, RonW thresholds were 2.83±0.15 log whereas RonB thresholds were 3.50±0.36 log. In BCM subjects, RonW thresholds were 4.52±0.43 log and RonB thresholds 6.11±0.55 log (Fig 3G). Substantially greater elevation (2.61 log) of RonB FST thresholds as compared to RonW FST (1.69 log) in BCM would be consistent with the greater efficacy of the blue background to suppress rod function. Consistent with this hypothesis, the difference between RonB and RonW thresholds in most BCM patients was similar to that expected from the desensitization of the scotopic system (Fig 3H). Notably, P27 and P29 appeared to be exceptions with their threshold differences close to that expected from the photopic system as it occurs in normal eyes.

### Location and Stability of Fixation in BCM

**Fig 4:**
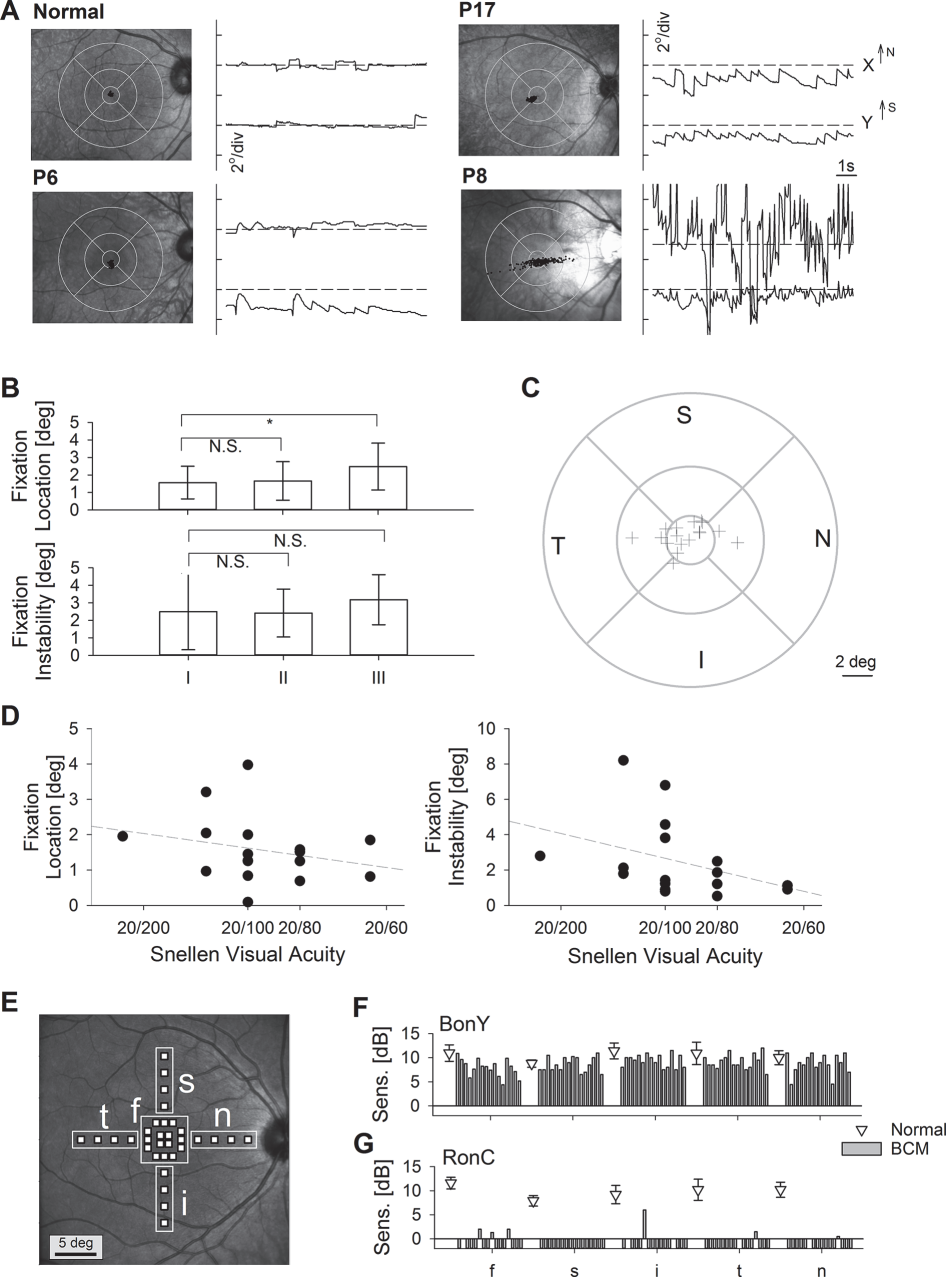
Nystagmus and foveal function in BCM. (A) Fixation locations in a normal subject and 3 BCM patients. For each subject, 10 s long epochs of eye movement data during fixation to a large visible red target (Target I) are shown in spatial (left) and spatio-temporal (right) coordinates. Spatial distribution of fixation clouds are shown on infrared SLO images of each macula with standard circles centered on the anatomical foveal depression. Spatio-temporal distribution of eye movements are shown on chart records for X and Y directions; up is nasal retina for X and superior retina for Y. All results are presented as equivalent right eyes for comparability. Horizontal dashed lines on the chart records depict the location of the anatomical fovea. (B) Fixation location and instability in BCM patients as a function of the bright red standard target (I), a green target (II) scotopically-matched to the standard target but expected to show greater visibility to S-cones, and a dim red target (III). N.S., not significant; *, P<0.05. (C) Distribution of fixation locations with the standard target in all patients. I = inferior, N = nasal, S = superior, and T = temporal retina. (D) Fixation location and instability as a function of best-corrected visual acuity. (E) Test pattern used with microperimetric stimuli to evaluate visual function under chromatic adaptation displayed on a normal near-infrared reflectance image. Stimulus locations are divided into 5 groups; f, foveal region, s, superior, i, inferior, t, temporal and n, nasal retina. (F,G) Sensitivities to blue stimuli on yellow background (BonY) and red stimuli on cyan background (RonC) in individual BCM patients (bars left to right; P2, P3, P4, P6, P8, P9, P10, P15, P16, P17, P18, P20, P25, P26, P28, and P29) compared to normal results (symbols; mean ±1sd) at the five regions shown in panel E. BCM results plotted below the zero line in Panel G represent those cases where the brightest available stimulus was not seen.

Congenital lack of normal L/M cone function in BCM leads to oculomotor abnormalties due to disrupted maturation of oculomotor circuits normally driven by sensory input from foveal L/M cones. BCM patients have involuntary oscillatory eye movements, called nystagmus, that result in instability of fixation. Normal subjects viewing a visible red target fixate steadily at the fovea, as determined under infrared imaging of the eye (Fig 4A). BCM patients viewing the same target show a range of oculomotor abnormalities from small (~1°) to large (>4°) amplitude nystagmus and average fixation locations ranging from fovea to parafovea (Fig 4A).

Across the BCM cohort, the average fixation location with a bright red target (Target I) was 1.6° eccentric from the anatomical foveal center, and the average fixation instability was 2.5° (Fig 4B). Next we questioned whether a green target (Target II) scotopically-matched to the standard target but with a greater visibility to S- and L/M-cones would change fixation characteristics. There was no significant change between the two targets in terms of location or instability (Fig 4B). Finally, with a dimmer red target (Target III), which was closer to the visibility threshold of rods, there was a significantly greater eccentricity of the fixation location to an average of 2.5°; fixation instability was not significantly changed (Fig 4B). The location of fixation with the standard Target I was mostly located near the anatomical fovea (Fig 4C); in a subset of 6 patients, the location was either in the temporal or nasal parafoveal region (Fig 4C). Best-corrected visual acuity in this cohort of BCM patients ranged from 20/63 (P3) to 20/200 (P7 and P25); median acuity was 20/100. Eyes with lower acuity tended to have greater fixation instability and tended to fixate further from the anatomical fovea (Fig 4D) but the slopes were not significantly different than zero (P>0.1).

### Foveal Function under Daylight Conditions with Chromatic Microperimetry

To better understand visual function at the anatomical fovea under light-adapted conditions, microperimetry was used with chromatic stimuli on chromatically-opposing adapting conditions in a subset of 16 BCM patients. A custom testing pattern was designed; there were 16 loci densely spaced within 2.2 degrees of the anatomical fovea, and 4 sets of 4 loci in the parafoveal regions along the major meridians (Fig 4E). With blue stimuli on yellow background (BonY), normal foveal sensitivity was 11.0±1.7 dB (Fig 4F). In BCM patients, BonY sensitivity was detectable but reduced to 7.9±1.8 dB (P<0.01). Parafoveally, BonY sensitivities were near 10 dB for both normal subjects and BCM patients; there were no significant differences between groups (Fig 4F). With red stimuli on a cyan background (RonC), normal foveal sensitivity was 11.6±1.2 dB (Fig 4G). Twelve of 16 BCM patients did not see the stimuli at the foveal locations implying a sensitivity loss of greater than 1 log unit. Four BCM patients (P2, P6, P10, and P15) did see the stimuli in 3–4 locations at a substantially reduced sensitivity of 1.3±0.9 dB. Notably one of these patients, P15, was previously shown to have spectral evidence of remnant L/M-cone function near the fovea [4]. Parafoveally, RonC sensitivities were near 9 dB for normal subjects. Twelve of 16 BCM patients did not see the stimuli parafoveally. The same four BCM patients with remnant RonC sensitivity at the fovea, showed one or more detectable loci in inferior, nasal and temporal parafovea; none of the BCM patients detected the RonC stimuli in the superior parafovea.

## Discussion

The X-linked retinal disease known as BCM, or X-linked incomplete achromatopsia [8], has a long history of clinical, electrophysiological and psychophysical investigation (e.g. [5–9]) and more recent studies of the molecular genetic basis of these cone opsin deficiencies (e.g. [2,13,33]). With the molecular genetic understanding of BCM and successful retinal gene therapy trials of other genetic conditions [39], we asked whether BCM could be a worthwhile target for gene augmentation therapy.

We began by determining whether there is sufficient L/M-cone structure in BCM to warrant gene augmentation therapy in humans [4], prompted by the successful treatment of color blindness in adult monkeys [40]. A cohort of BCM patients with large deletion mutations involving L/M-cone pigment genes and/or their upstream regulatory elements were studied in detail by optical imaging methods, and evidence was provided for a reduced number of residual L/M cone cells with abnormal but detectable outer segments in the central retina. It was concluded that there could be potential value in gene therapy of BCM, but the fragile central retinal structure in BCM would make a subfoveal injection a less attractive choice than intravitreal delivery [4]. The limitation of intravitreal delivery in a retina-wide cone disease is that the only retinal area receiving treatment is likely to be the foveal region [41,42].

Despite the long history of observations in BCM, do we understand sufficiently the phenotype of BCM using instruments and techniques currently available in most clinics to devise outcome measures for an early phase clinical trial? Safety parameters, the primary outcomes, would monitor for systemic as well as ocular toxicity, and would include the routine ophthalmic examination and OCT [43]. The latter method would also be used for pre-enrollment imaging for selection of candidates with detectable foveal outer nuclear layer and cone outer segment structure [4]. Secondary outcomes for efficacy would include, of course, measurement of best-corrected visual acuity. Electroretinography was not considered as an outcome measure in this study because the method would not be sensitive enough to measure focal retinal function changes in a trial where only a small region of the retina will be treated.

The current study was of BCM patients with large *OPN1LW* and *OPN1MW* gene array deletion mutations, a limited number with the common C203R point mutation and one with a single red-green opsin gene and the C203R point mutation [10,16,33]. There were sufficiently similar functional phenotypes in this cohort of patients to make recommendations for efficacy outcomes that could detect L/M cone improvement.

A standard method of isolating cone function is with the use of a background light that is just bright enough to substantially desensitize the rod vision but only minimally affect cone vision [44,45]. The background chosen for most clinical perimeters is a homogeneous white light at a luminance of 10 cd.m^-2^ (31.5 asb). Spectral sensitivity measurements in BCM patients (Fig 2) showed that middle- and long-wavelength increments presented on this standard background light are mediated by the rod system; shorter wavelength stimuli are seen either by the S-cone system or the rod system, depending on the retinal location and specific patient. The standard 10 cd.m^-2^ background is the luminance of a white paper in typical indoor lighting, and it is somewhat counterintuitive to observe retained night vision under day vision conditions in BCM. However, the standard background is more than a log unit dimmer than the luminance when rod saturation is expected to set in [35]. The existence of functioning rods (in addition to S-cones) likely explains the normal or nearly normal kinetic perimetry extents in the BCM patients. Taken together with the known variability of this test in other inherited retinopathies [46,47] it can be concluded that kinetic perimetry (with Goldman V stimuli and standard background) is undesirable as an outcome of L/M-cone efficiency in BCM. Unlike kinetic perimetry, the results with computerized static perimetry with white and 600-nm stimuli were significantly abnormal in all BCM patients (Fig 1). Use of relative spectral effectiveness values together with individual estimates of S-cone sensitivities [48] suggested that light-adapted static perimetry with white stimuli was likely driven by the combination of rods and S-cones, whereas visibility of 600-nm stimuli was dominated by rod function. If light-adapted white static perimetry were to be used as an efficacy outcome, large (~1 log) increases in sensitivity resulting in normalization of thresholds could be evidence of L/M cone function, but smaller increments would likely require additional spectral sensitivity measures to differentiate L/M-cone origins from rod systems such as it would be obtained by squinting.

Under dark-adapted conditions in normal eyes, rod sensitivity far exceeds that of cones and dominates function across the retina independent of wavelength. One exception to this is a small region at or near the fovea, where the dramatic local increase in L/M cone density together with the decrease in rod density [49] results in a ‘mixed’ spectral sensitivity function where longer wavelength lights are seen by L/M-cones and shorter and middle wavelength lights are seen by rods [50]. BCM patients under dark-adapted conditions in the central retina showed large losses of sensitivity at longer wavelengths but not at middle wavelengths; the mediation was by rods. This type of two-color dark-adapted threshold testing at the central retina can be thus considered as an outcome measure (assuming foveal L/M-cone targeting from intravitreal injection) where an L/M-cone based improvement would be expected to improve the long-wavelength thresholds while the middle-wavelength thresholds remained unchanged. Similarly, we introduced a novel chromatic microperimetry method whereby visual function at the anatomical fovea was tested under real-time retinal tracking. Results showed normal BonY sensitivity likely representing a dominant component from S-cone function and substantial loss of RonC sensitivity corresponding to the loss of L/M-cone function.

Full-field stimulus testing (FST) with chromatic stimuli under dark-adapted conditions allows detection of the highest sensitivity within the retina for a given stimulus [18,29] independent of fixation and it has been used as an outcome measure in a clinical trial of gene therapy [43]. The next advance was chromatic FST on increasingly brighter backgrounds in order to distinguish rod and cone function in patients in whom dark-adapted results are rod mediated [25]. Here we extended the FST method one step further by using a range of chromatic backgrounds in order to preferentially suppress receptors sensitive to shorter or longer wavelengths. In the dark-adapted state, BCM patients had normal and rod-mediated thresholds; and this was expected. Blue thresholds under yellow or white light adaptation were shown to be dominated by S-cone function both in normal subjects and BCM patients. Red thresholds under blue or white light adaptation, however, were severely abnormal in BCM; spectral effectiveness of the adapting background suggested mediation by rods. If red FST on chromatic backgrounds were to be used as efficacy outcomes, an improvement of L/M-cone function independent of the retinal location should be detectable.

By clinical observation, nystagmus could range from mild to severe in BCM patients and has been reported to become not noticeable in some patients with age [2,6,10,12–15,51]. Rarely, analyses of eye movement recordings have shown pendular nystagmus of various amplitudes [52], and location of fixation, when quantified, was found to be foveal or parafoveal [8,28]. Since in some conditions eye movement characteristics are strongly influenced by visual feedback [53,54], eye movement recordings in BCM in the current study were performed under careful control of the visual environment. Eyes were dark-adapted, and patients were directed to fixate to a single stationary visible target in darkness; fixation location and stability were recorded under infrared view of the retina. Oculomotor abnormalities were found with pendular as well as jerk components along horizontal, diagonal and vertical directions; amplitude of eye movements could span a wide range and the average location of fixation could be centered on the fovea or in the parafovea. Quantifiable recordings such as performed here would be very important to obtain before and after treatments directed to the foveal region since possible increases in L/M cone sensitivity could cause changes to the fixation location [32,55] and to fixation stability.

In conclusion, we have provided data on a range of localized and full-field measures of visual function in a group of BCM patients. Further studies need to define the variability of a subset of these measures that could be used as outcomes in future BCM treatment trials.

## Supporting Information

S1 Fig Cross-sectional OCT scans and ONL analyses along the vertical meridian in a subset of BCM patients not previously published. (A) Normal scan compared with those of three BCM patients. ONL is highlighted in blue. (B) ONL thickness is graphically displayed for a group of normal subjects (gray, mean±2SD; n = 22; ages 8–62 years) and from 8 BCM patients (ages 13–72). Patient data are lines and are comparably presented as in Fig 2C of reference 4. The thinnest ONL profile is from P22, the 72-year-old patient (arrow).
(PDF)

S1 Table Clinical characteristics of the BCM patients. (PDF)
