# Supplementary figures and images for: Blue Cone Monochromacy: Visual Function and Efficacy Outcome Measures for Clinical Trials
Source: PLoS One. 2015 Apr 24;10(4):e0125700. doi: 10.1371/journal.pone.0125700 (PMC4409040; doi:10.1371/journal.pone.0125700)

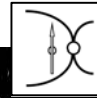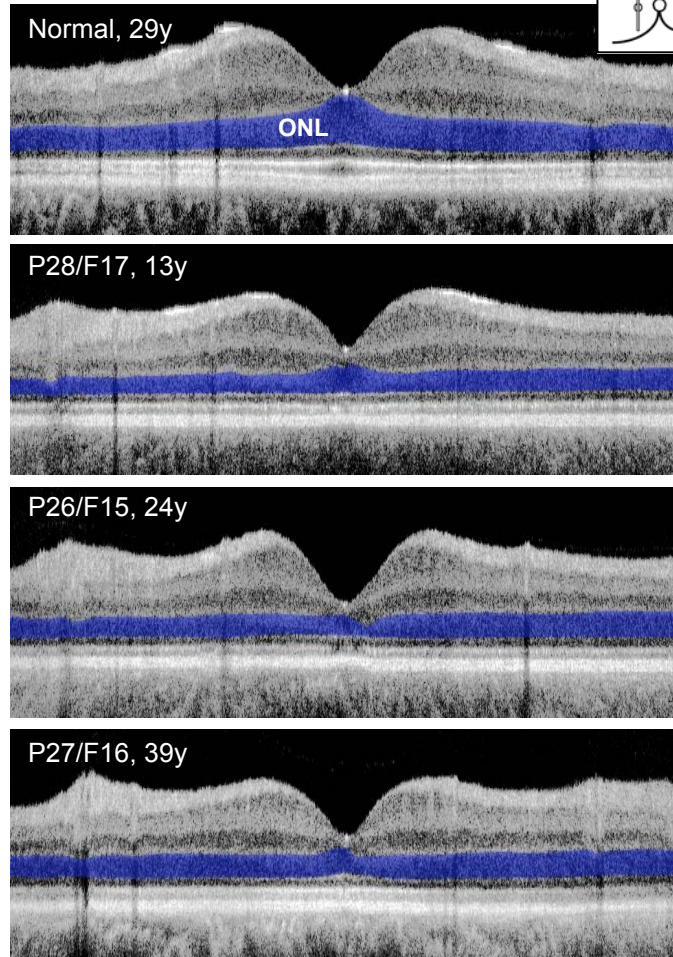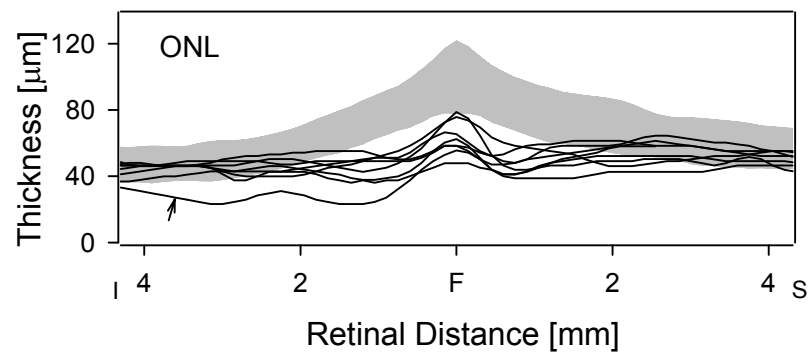

Supplement: S1 Fig — (A) Normal scan compared with those of three BCM patients. ONL is highlighted in blue. (B) ONL thickness is graphically displayed for a group of normal subjects (gray, mean±2SD; n = 22; ages 8–62 years) and from 8 BCM patients (ages 13–72). Patient data are lines and are comparably presented as in Fig 2C of reference 4. The thinnest ONL profile is from P22, the 72-year-old patient (arrow). (PDF) [file pone.0125700.s001.pdf]
