# Supplementary material for: Blue Cone Monochromacy: Visual Function and Efficacy Outcome Measures for Clinical Trials
Source: PLoS One. 2015 Apr 24;10(4):e0125700. doi: 10.1371/journal.pone.0125700 (PMC4409040; doi:10.1371/journal.pone.0125700)
Supplement: S1 Table — (PDF) [file pone.0125700.s002.pdf]

S1 Table. Clinical characteristics of the BCM patients

Patient/ Family[a]	Age at visits (y)	Mutation class: mutation detail	Visual acuity[b]	Refraction[c]
P1/F1	5,8	Deletion: *OPN1LW*, *OPN1MW* (partial)	20/100	-12.50
P2/F1	8,10	Deletion: *OPN1LW*, *OPN1MW* (partial)	20/80	-3.75
P3/F1	11,13	Deletion: *OPN1LW*, *OPN1MW* (partial)	20/63	-12.75
P4/F1	48,50	Deletion: *OPN1LW*, *OPN1MW* (partial)	20/100	-10.25
P5/F2	7	Deletion: LCR, *OPN1LW* (partial)	20/100	-3.25
P6/F2	12	Deletion: LCR, *OPN1LW* (partial)	20/100	-5.00
P7/F3	5,7	Deletion: LCR, *OPN1LW* (partial)	20/400[d]-20/200	-0.50
P8/F3	10,19	Deletion: LCR, *OPN1LW* (partial)	20/100	-5.00
P9/F3	16,25	Deletion: LCR, *OPN1LW* (partial)	20/100	-6.00
P10/F3	19,28	Deletion: LCR, *OPN1LW* (partial)	20/125	-6.50
P11-P14[e]				
P15/F6	14	Deletion: LCR, *OPN1LW* (partial)	20/100	-4.00
P16/F7	28	Deletion: LCR, *OPN1LW* (partial)	20/125	-8.50
P17/F8	33	Deletion: LCR, *OPN1LW* (partial)	20/100	-3.75
P18/F9	35	Deletion: LCR, *OPN1LW* (partial)	20/63	-5.50
P19/F10	43	Deletion: LCR, *OPN1LW* (partial)	20/80	-6.00
P20/F11	55	Deletion: LCR, *OPN1LW* (partial)	20/80	-6.50
P21/F12	18	Deletion: *OPN1LW, OPN1MW*	20/160-20/100	-8.50
P22/F12	72	Deletion: *OPN1LW, OPN1MW*	20/125-20/100	-3.00
P23/F13	30	Deletion: LCR	20/100	-10.00
P24/F14	27	Deletion: LCR	20/100-20/125	-8.50
P25/F15	18	Deletion: LCR, *OPN1LW* (partial)	20/200-20/125	-11.25
P26/F15	24	Deletion: LCR, *OPN1LW* (partial)	20/70-20/80	-9.25
P27/F16	39	Missense: p.C203R in a single red-green hybrid gene	20/80	-1.00
P28/F17	13	Missense: p.C203R	20/80	-5.50
P29/F17	35	Missense: p.C203R	20/100	-5.25

OPN1LW, long-wave-sensitive opsin-1 gene; *OPN1MW*, medium-wave-sensitive opsin-1 gene; LCR, locus control region.

[a]Patients P1-P20 have been described previously [4].

[b]Best corrected visual acuity at most recent visit; similar in the two eyes; otherwise, specified individually, as RE-LE.

[c]Refraction at most recent visit; spherical equivalent; average of both eyes.

[d]Eye with macular coloboma.

[e]Patients P11-P14 are not included because detailed visual function measurements were not performed.
